# Renal AL Amyloidosis: Updates on Diagnosis, Staging, and Management

**DOI:** 10.3390/jcm13061744

**Published:** 2024-03-18

**Authors:** Areez Shafqat, Hassan Elmaleh, Ali Mushtaq, Zaina Firdous, Omer Ashruf, Debduti Mukhopadhyay, Maheen Ahmad, Mahnoor Ahmad, Shahzad Raza, Faiz Anwer

**Affiliations:** 1 College of Medicine, Alfaisal University, Riyadh 11533, Saudi Arabia; ashafqat@alfaisal.edu; 2 Department of Hematology and Medical Oncology, Cleveland Clinic Taussig Cancer Institute, Cleveland, OH 44195, USA; elmaleh@ccf.org (H.E.); razas@ccf.org (S.R.); 3 Department of Internal Medicine, Cleveland Clinic Foundation, Cleveland, OH 44195, USA; mushtaa2@ccf.org; 4 Department of Hospital Medicine, WellSpan York Hospital, York, PA 17403, USA; drzaina.f@gmail.com; 5 College of Medicine, Northeast Ohio Medical University, Rootstown, OH 44272, USA; oashruf@neomed.edu; 6 Department of Medicine, Jacobs School of Medicine, State University of New York at Buffalo, Catholic Health System, Buffalo, NY 14260, USA; debdutim@buffalo.edu; 7 Department of Molecular, Cellular, & Developmental Biology, University of California, Santa Barbara, CA 93106, USA; 8 Department of Human Biology and Society, University of California, Los Angeles, CA 90095, USA

**Keywords:** AL amyloidosis, daratumumab, venetoclax, immunotherapy, anti-amyloid antibodies

## Abstract

AL amyloidosis is caused by the excessive production of nonfunctional immunoglobulins, leading to the formation of amyloid fibrils that damage vital organs, especially the heart and kidneys. AL amyloidosis presents with non-specific symptoms such as fatigue, weight loss, numbness, pain, and nephrotic syndrome. Consequently, diagnosis is often delayed, and patients typically present with advanced disease at diagnosis. The Pavia renal staging model stratifies patients based on their likelihood of progressing to dialysis. Treatment with daratumumab plus cyclophosphamide, bortezomib, and dexamethasone (i.e., Dara-CyBorD) was effective in inducing renal response in the landmark phase III ANDROMEDA trial and reducing early mortality. However, determining the most appropriate treatment regimen for relapsed or refractory cases remains a challenge due to various patient- and disease-related factors. Encouragingly, t(11:14) may be a positive indicator of therapy responses to the anti-BCL2 therapy venetoclax. Moreover, it is increasingly possible—for the first time—to clear AL amyloid fibrils from peripheral organs by leveraging novel anti-fibril immunotherapeutic approaches, although these medications are still under investigation in clinical trials. Given these advancements, this review provides a comprehensive overview of the current strategies for diagnosing, staging, treating, and monitoring AL amyloidosis, emphasizing renal involvement.

## 1. Introduction

Immunoglobulin light chain (AL) amyloidosis arises from a plasma cell dyscrasia that results in the overproduction of nonfunctional immunoglobulins [1]. These misfolded proteins aggregate into insoluble β-pleated amyloid fibrils, commonly known as AL amyloids, which deposit within various organs. Cardiac and renal damage are most prevalent among patients with AL amyloidosis [2,3]. Renal involvement, leading to nephrotic syndrome, occurs in about two-thirds of AL amyloidosis cases, with 25% of these patients progressing to end-stage renal disease (ESRD) and requiring renal replacement therapy, including dialysis and kidney transplantation [4,5]. 

Currently, the goal of treatment is to reduce amyloid production by targeting the aberrant plasma cell clone in the bone marrow [3,6]. However, the prospect of clearing peripheral amyloid deposits with the novel anti-fibril antibodies looks promising, through approval of these medications is still subject to ongoing clinical trials [7]. Ongoing Phase III studies testing the efficacy of these anti-fibril medications focus on advanced cardiac amyloidosis. Novel imaging techniques applied to the early diagnosis of AL amyloidosis are also primarily concentrated on cardiac involvement, given its importance to patient prognosis. In contrast, renal patients, who also often present with advanced disease at diagnosis reflecting delayed or incorrect diagnoses, are relatively underrepresented in such studies. Furthermore, biomarkers of renal involvement are limited to conventional renal functional tests such as estimated glomerular filtration rate (eGFR) and measures of proteinuria. This paper provides an overview of the diagnosis, staging, and management of renal AL amyloidosis while highlighting prevailing challenges that future studies must address. 

## 2. Diagnostic Approach

Patients with AL amyloidosis exhibit a non-specific symptom profile, which may include fatigue, unintentional weight loss, arrhythmia, numbness, paresthesia, pain, enlarged tongue (macroglossia), and nephrotic syndrome. Consequently, the diagnosis of AL amyloidosis is often delayed, with the median time from symptom onset to diagnosis potentially extending between 2 and 4 years [8]. Indeed, approximately 37% of patients are diagnosed over 12 months post symptom onset, with 32% consulting at least five doctors before receiving a diagnosis [9]. This delay leads to irreversible organ failure at presentation, including progressive heart failure, ESRD, and death. 

The consequences of renal involvement range from mild proteinuria to nephrotic-range proteinuria, its associated manifestations (hyperlipidemia, peripheral edema, hypercoagulability, and increased susceptibility to infections), and progressive renal dysfunction [10]. Timely recognition and the prompt initiation of treatment facilitate the preservation of organ function, mitigating dysfunction and improving overall survival [11]. 

For a diagnosis of AL amyloidosis, there must be evidence of an amyloid-related syndrome, positive Congo Red staining on biopsy (or detection of AL amyloid on mass spectrometry), and the presence of a plasma cell dyscrasia. The initial step in diagnosing AL amyloidosis involves detecting circulating monoclonal light chains (Table 1). Serum protein electrophoresis with immunofixation, 24 h urine protein collection for electrophoresis with immunofixation, and serum-free light chain (FLC) assay can detect amyloidogenic monoclonal components effectively. The absence of a monoclonal component renders the diagnosis of AL amyloidosis unlikely [12]. Amyloid typing is a critical step, and methods include immunofluorescence (IF), immunohistochemistry (IHC), electron microscopy (EM), immunoelectron-microscopy (IEM), and laser microdissection with tandem mass spectroscopy (LMD-MS)-based proteomic analysis [13,14,15,16]. Commonly sampled surrogate biopsy sites include the abdominal fat pad, bone marrow, and minor salivary glands. At referral centers, the diagnostic sensitivity of abdominal fat pad biopsy is 70–80%, that of bone marrow biopsy is 70%, and that of the minor salivary gland is 80% [17]. Simultaneously sampling the abdominal fat and bone marrow can increase sensitivity to 89% [18]. 

In clinical practice, the detection of amyloid in a bone marrow biopsy is not uncommon, and the marrow biopsy additionally reveals the underlying plasma cell dyscrasia or lymphoproliferative disease. Fluorescence in situ hybridization (FISH) on a bone marrow biopsy can identify cytogenetic abnormalities that carry significant prognostic and therapeutic ramifications. Positive findings of marrow and surrogate site biopsy often obviate the need for an organ (i.e., renal or cardiac) biopsy. However, if surrogate site biopsy yields negative results, an organ biopsy should be expedited for possible AL amyloidosis. 

Conventional contraindications to a kidney biopsy, both relative (e.g., solitary kidney and CKD) and absolute (e.g., uncontrolled bleeding disorder with hypertension, active skin infection over the biopsy site, pyelonephritis, and patient inability to tolerate the procedure), apply when deciding to perform a kidney biopsy. Notably, in the absence of a hemostatic disorder, renal AL amyloidosis does not independently increase the risk for bleeding secondary to a renal biopsy, in contrast to a liver biopsy in cases of hepatic amyloidosis, which exhibits an increased bleeding risk [19]. 

The differential diagnosis of renal involvement, proteinuria, and monoclonal gammopathy of undetermined significance (MGUS) includes AL amyloidosis and comorbidities such as diabetes. The monoclonal gammopathy of renal significance (MGRS) encompasses a spectrum of disorders arising from the deposition of monoclonal immunoglobulin, including but not limited to myeloma cast nephropathy, light-chain proximal tubulopathy, monoclonal immunoglobulin (Ig) deposition disease, immunotactoid glomerulopathy, Fanconi syndrome, fibrillary glomerulonephritis, and membranous-like glomerulopathy with masked IgG κ deposits [20,21]. A kidney biopsy allows for renal pathological diagnosis and facilitates the determination of the mechanism of injury and the exploration of treatment options. 

Addressing the unmet need to diagnose AL amyloidosis early on is crucial for improving patient survival and quality of life. Educating medical students and practicing physicians on AL amyloidosis is a necessary initial step to bridge the existing education gap. Regarding imaging approaches, the utilization of the amyloid-reactive antibody AT-01 for detecting systemic amyloid deposits on Positron Emission Tomography (PET)/Computed Tomography (CT) imaging has reported notably high sensitivity for cardiac amyloidosis (positive percent agreement (PPA) of 96.2% and negative percent agreement (NPA) of 100%) but less for the kidney (PPA = 78.6% and NPA = 80%) [22]. Other advanced imaging techniques—e.g., [^18^F]florbetapir PET/CT imaging—have shown the ability to detect cardiac amyloid deposition in 50% of patients deemed to have no cardiac and other organ (e.g., tongue, lungs, parotid gland) involvement based on conventional criteria (echocardiographic and biomarker), with excellent interobserver reproducibility and interobserver repeatability [23,24,25]. However, the results were less favorable regarding kidney and abdominal wall fat involvement [25]. 

Technicum-99m (^99m^Tc)-based scintigraphy—such as ^99m^Tc-labelled pyrophosphate (^99m^Tc-PYP)/3,3-diphosphono-1,2-propanodicarboxylic acid (^99m^Tc-DPD)/hydroxy methylene diphosphonate (^99m^Tc-HMDP) tracers—has been evaluated for detecting cardiac amyloidosis. These techniques have a high sensitivity (92–99%) and specificity (86–92%) in patients with transthyretin (ATTR) cardiac amyloidosis and form the basis of the radionuclide bone scintigraphy-based non-biopsy diagnostic criteria (NBDC) of cardiac ATTR amyloidosis, which requires a clinical phenotype of cardiomyopathy, evidence of cardiac infiltration on echocardiography or CMR, the absence of monoclonal gammopathy, and a grade 2–3 radionuclide scan using ^99m^TC-PYP/DPD/HMDP to establish a diagnosis of ATTR cardiomyopathy, without the need for endomyocardial biopsy [26,27,28]. AL amyloidosis is a common cause of false positives when cardiac tracer uptake is observed, and, consequently, a monoclonal component or light chain abnormalities must be screened for by serum and urine immunofixation and FLC assays upon any myocardial radiotracer uptake. Once a monoclonal component has been excluded, a diagnosis of ATTR cardiomyopathy can be confirmed [29]. A grade 0 radionuclide scan almost excludes ATTR amyloidosis, and most cases are due to AL cardiac amyloidosis. CKD can affect serum FLC levels, and refining FLC cut-offs in these patients further improves the diagnostic performance of the NBDC algorithm [30].

## 3. Staging and Risk Stratification

The staging models utilized for AL amyloidosis include the Mayo 2004, Mayo 2004 with European modification, and Mayo 2012 systems. These models are primarily based on the extent of cardiac involvement—the most important predictor of overall survival in AL amyloidosis (Table 2). 

While the impact of renal involvement on overall survival may be less prominent than cardiac involvement, the existing renal staging models provide robust predictions about the patient’s risk of advancing to ESRD (Table 3) [31,35,36]. The Pavia renal staging model stratifies patients based on their risk of progressing to dialysis, with the proportion of patients requiring dialysis within three years after diagnosis ranging from 0–4% in renal stage I to 60–85% in renal stage III [36]. This further underscores the need for early diagnosis and treatment before organ damage ensues [36]. Renal survival in this model, defined as the duration between diagnosis and the initiation of dialysis, is significantly influenced by factors like proteinuria and eGFR. However, there is no substantial difference in overall survival among the three renal stages [36]. Renal staging models incorporating the 24 h proteinuria (UPr) to eGFR ratio (UPr/eGFR ratio) categorize patients into three renal stages: Stage 1 with a ratio of <30, Stage 2 with a ratio between 30 and 99, and Stage 3 with a ratio of ≥100. At the 3-year landmark, the progression rates to dialysis for patients in stages 1, 2, and 3 were 0%, 11%, and 46%, respectively [37]. Considering the limitations of the 24 h proteinuria measurement, including its potential inaccuracy and cumbersome collection process, a staging system employing the more straightforward urinary albumin/creatinine ratio (UACR) test has recently been introduced [38,39]. Aside from its convenience, incorporating UACR may offer superior discrimination between renal stages and an improved prediction of renal response to therapy [38,39]. 

## 4. Assessment of Therapy Response

The treatment response in AL amyloidosis can be assessed at the hematological and organ level. Hematological response evaluation revolves around quantifying serum-FLCs, where a reduction in FLCs is the strongest predictor of overall survival in AL amyloidosis [40]. The criteria for different levels of hematological response are detailed in Table 4. The achievement of at least (≥) a very good partial response (VGPR) is associated with significantly better overall and organ survival. In cases where hematological complete response (CR) is achieved, the assessment of minimal residual disease (MRD) through next-generation flow cytometry (NGF) or next-generation sequencing (NGS) can be used to detect residual amyloidogenic clonal plasma cells. MRD negativity is associated with longer progression-free survival, a reduced probability of hematological relapse, and an increased likelihood of organ response [41]. 

For patients with a low dFLC burden (<50 mg/L), a reduction in dFLC of <10 mg/L post-treatment signifies a response and correlates with improved overall and renal survival (i.e., extended time to dialysis) [42]. This subgroup of patients often exhibits a higher prevalence of renal involvement and more pronounced proteinuria. However, cardiac and other organ involvements are less frequent and severe than those with dFLC ≥ 50 mg/L [43].

Organ response assessment is an essential factor for outcome prediction in AL amyloidosis. Biomarkers for cardiac (NT-proBNP, troponin), renal (eGFR, proteinuria), and liver (ALP) are used to evaluate organ response (Table 4) [44]. As opposed to a binary organ response assessment model (response or no response), graded models have been validated for cardiac and renal responses [45,46] (Table 5). These models—like the renal staging and progression models—are predictive of the likelihood of advancing to dialysis rather than mortality. Achieving VGPR or better is associated with improved renal survival [36]. Importantly, renal responses may appear 12–24 months later than hematologic responses or, like cardiac responses, may occur gradually throughout therapy. Routine clinical practice involves evaluating hematologic and organ responses every 1–2 cycles during active treatment. These assessments are conducted three months post-autologous stem cell transplantation (ASCT) and every three months after [7]. 

## 5. Treatment of AL Amyloidosis

### 5.1. Induction Therapy

The first-line induction therapy for individuals diagnosed with AL amyloidosis is the combination of daratumumab, cyclophosphamide, bortezomib, and dexamethasone (Dara-CyBorD), established as the standard of care based on the ANDROMEDA trial [47]. The results demonstrated a significantly higher likelihood of achieving a hematological CR in the Dara-CyBorD group over a median follow-up of 11.4 months, along with a lower risk of reaching a composite surrogate secondary endpoint of organ (cardiac or renal) deterioration, hematologic progression, or death [47]. Dara-CyBorD achieved a renal response in 53% of patients at 6 months, with only 4.3% progressing, while the control group had 23.9% achieving a renal response and 11.5% suffering renal progression. A recent retrospective study revealed that 73.1% of patients with more advanced cardiac involvement than those in the ANDROMEDA trial rapidly achieved ≥ VGPR, with a cardiac response rate of 58.3% [48]. In the pre-daratumumab era, early 6-month mortality for AL amyloidosis reached 25% [49,50], whereas Dara-CyBorD induction therapy resulted in a 6-month early mortality rate of 7.6% [48]. However, it should be noted that ANDROMEDA did not include patients with stage IIIB cardiac amyloidosis, who are at highest risk for early mortality. Nevertheless, the introduction of daratumumab has thus marked a transformative advance in AL amyloidosis care.

Intravenous (I/V) daratumumab has demonstrated reduced efficacy in patients with nephrotic-range albuminuria with an albumin-to-creatinine ratio (ACR) > 220 mg/mmol and dFLC ≥ 180 mg/L. This diminished efficacy may be attributed to the loss of daratumumab in the urine of nephrotic patients, leading to insufficient serum concentrations [51,52]. Future trials must explore this effect, as the ANDROMEDA study did not compare nephrotic versus non-nephrotic patients. Subcutaneous (SC) daratumumab—currently widely used in North America owing to its lower rates of infusion-related reactions and shorter administration times—may be more effective in these patients [53,54]. 

### 5.2. Treatment of Relapsed or Refractory AL Amyloidosis

Refractory AL amyloidosis describes hematologic or organ progression or stable disease despite ongoing therapy. Failure to achieve a hematologic response within 2 months or ≥VGPR within 4 months of treatment is deemed a refractory disease. Patients who achieve hematological CR to induction therapy and renal responders demonstrate lower rates of disease relapse post-ASCT, and those with relapsed disease who responded to initial therapy exhibit significantly better survival outcomes compared to those refractory to induction treatment [43,55]. 

Patients with relapsed or refractory AL amyloidosis should be offered alternative therapies. However, determining the most appropriate regimen presents challenges, given the multiple patient- and disease-related factors. The selection process must account for factors such as the depth and duration of the initial response, prior treatments, patient and physician preferences, the patient’s performance status, the presence and extent of end-organ damage, anticipated treatment-related toxicity, drug availability, and insurance coverage [56,57]. A consensus is lacking regarding the optimal timing for initiating second-line therapy in cases of progressive disease. One approach recommends early intervention, particularly for individuals with “high-risk dFLC” (defined as a dFLC of >20 mg/L, a level >20% of the baseline value, and a >50% increase from the value reached at best response), without waiting for cardiac and renal progression [55]. Conversely, an alternative strategy suggests delaying treatment until organ dysfunction becomes evident. This approach is supported by the prolonged interval between hematologic progression and organ progression or relapse, sometimes reaching up to 100 months. Consequently, delaying the second-line treatment can preserve patients’ quality of life for months and spare them from potential treatment-related adverse effects [58].

Daratumumab, whether as a monotherapy or as part of a combination regimen, is increasingly used in relapsed/refractory AL amyloidosis. Observational studies have indicated that daratumumab can induce early and deep hematological and organ responses in this patient population (reviewed here [59]). Two prospective phase II trials (NCT02841033 and NCT02816476) have reported hematological VGPR or better in 47.6–86% of cases and a renal response in 67% of relapsed AL amyloidosis patients receiving I/V daratumumab monotherapy [60,61]. Among patients who achieve ≥VGPR, daratumumab monotherapy induces MRD negativity in 47% of cases, indicating sustained responses [62]. In a subset of 10 patients given I/V daratumumab as maintenance, 6 exhibited sustained MRD negativity, 2 showed persistent MRD positivity, and 2 transitioned from MRD-positive to negative status [62]. In a separate group of patients that completed I/V daratumumab therapy and were followed up at 12-month intervals with MRD testing, 39% maintained MRD negativity, 43% exhibited persistent MRD positivity, 2% achieved MRD clearance after 17 months, and 2% experienced MRD resurgence 26 months post-daratumumab cessation [62]. These findings advocate for the use of daratumumab in relapsed/refractory AL amyloidosis. 

The most effective myeloma drugs to combine with daratumumab—such as immunomodulatory drugs (IMiDs, e.g., lenalidomide) or proteasome inhibitors (e.g., bortezomib)—remain uncertain. Daratumumab–bortezomib, when administered to bortezomib-naïve patients, resulted in 66% of patients achieving ≥VGPR after three months of treatment initiation [51]. For patients who received upfront bortezomib or those with severe polyneuropathy, daratumumab–lenalidomide yielded an overall response rate of 84% after three months of treatment initiation [63]. Importantly, lenalidomide is predominantly excreted through urine and, therefore, requires dosage adjustments in cases of renal impairment, potentially limiting its use in renal patients. The selection of an appropriate daratumumab-based combination regimen for patients previously exposed to daratumumab during induction remains inadequately studied. A recent study combining daratumumab, pomalidomide, and dexamethasone (DPd) in nine patients with relapsed AL amyloidosis who had received at least eight prior doses of daratumumab showed rapid and deep hematological responses in all five evaluable cases [64]. Regarding renal response, five out of six patients with assessable data exhibited positive organ responses. In three patients who continued DPd beyond 12 cycles, hematological and organ responses were sustained beyond 26 months [64].

The t(11:14) translocation is the most prevalent cytogenetic abnormality in AL amyloidosis. t(11:14) is an adverse prognostic factor associated with a higher likelihood of cardiac involvement and a negative predictor of hematologic and organ responses to proteasome inhibitor therapies such as bortezomib [65,66]. Importantly, however, t(11:14) is a positive predictor of response to the anti-BCL2 agent venetoclax in multiple myeloma [67]. A retrospective study using the anti-BCL2 drug venetoclax in relapsed/refractory t(11;14) AL amyloidosis demonstrated a hematological response rate of 68% and a renal response of 40% [68]. A multicenter retrospective study reported a hematological response rate of 88%, albeit with a lower renal response of 15% [69]. Another retrospective study achieved ≥VGPR in 78% of patients with relapsed/refractory t(11;14)-positive AL amyloidosis with venetoclax-based therapy [68]. Venetoclax-based regimens can also be considered for t(11:14)-positive AL amyloidosis patients with inadequate responses to daratumumab-based treatment [70]. In a study by Orland et al., venetoclax-based regimens resulted in a rapid hematologic response of VGPR or better in 86% of daratumumab-exposed relapsed/refractory AL amyloidosis patients with only two cycles of therapy [71]. 

The B-cell maturation antigen (BCMA) expressed on plasma cells is an excellent target for cellular and immunotherapies in multiple myeloma and AL amyloidosis [72]. Various drugs targeting BCMA-expressing plasma cells include the antibody-drug conjugate Belantamab–Mafodotin, CAR T cells, and bispecific antibodies/bispecific T-cell engagers [72,73]. Three retrospective studies on Belantamab–Mafodotin monotherapy—involving 45 relapsed AL amyloidosis patients—demonstrated a hematologic overall response rate of 75–80% and ≥VGPR of 64–66% [74,75,76]. These response rates surpass those observed in multiple myeloma, suggesting that AL amyloidosis may either feature a lower burden of plasma cell clones than multiple myeloma or harbor a clone that may be more sensitive to anti-BCMA-directed therapy than myeloma [75]. Ocular toxicity, particularly keratopathy, stands out as a notable side effect of Belantamab–Mafodotin, potentially limiting dosage and necessitating drug discontinuation. An ongoing phase 2 clinical trial (NCT04617925) is assessing the safety of Belantamab–Mafodotin monotherapy in patients with relapsed or refractory AL amyloidosis. 

Two CAR T-cell therapies (Abecma and Carvykti) have been approved to treat relapsed/refractory multiple myeloma [77,78]. Oliver-Caldes et al. reported the first experience of using anti-BCMA CAR T-cell therapy in a patient with multiple myeloma who developed renal AL amyloidosis, inducing sustained complete hematological remission at 12 months with a 70% decrease in proteinuria [79]. The anti-BCMA CAR T cell HBI0101 showed overall tolerability in four relapsed/refractory AL amyloidosis patients (NCT04720313), with manageable grade 3 cytokine release syndrome in two patients and no observed neurotoxicity [80]. Although it would be premature to draw any conclusions about HBI0101’s efficacy, all four patients—of which three had stage IIIA cardiac AL amyloidosis—achieved CR and demonstrated evidence of an organ response [80]. 

Teclistamab, a BCMA x CD3 bispecific T-cell engager, has recently been approved by the FDA for the treatment of relapsed and refractory multiple myeloma, achieving an overall response rate of 63% and MRD negativity in 26% of patients [81]. In a retrospective case series involving three patients with relapsed/refractory AL amyloidosis and concurrent multiple myeloma, a hematologic response was observed in two patients, including one CR and one PR, within 3 weeks of treatment initiation [82]. Another multinational retrospective cohort study of 17 relapsed/refractory AL amyloidosis patients, most of whom were exposed to daratumumab-based therapies, demonstrated a VGPR or better in 88% of patients with teclistamab [83]. Importantly, no cardiac or renal toxicity was observed; however, 29% of patients suffered from infections, leading the authors to recommend prophylactic measures during teclistamab therapy [83]. Clinical trials on bispecific antibodies/bispecific T-cell engagers targeting BCMA in AL amyloidosis are awaited. 

### 5.3. Anti-Amyloid Fibril Antibodies

CAEL-101 (formerly mAb11-F14) is an IgG1 monoclonal antibody that binds AL amyloid fibrils, facilitating amyloid clearance via opsonization, i.e., facilitating phagocytosis by monocytes/macrophages and neutrophils via engagement with the Fcγ receptor [84,85]. Phase 1 and 2 clinical trials have established the long-term safety profile and tolerability of CAEL-101 in AL amyloidosis patients [86,87,88,89]. A phase II dose-finding study determined that CAEL-101 can be administered at dosages of up to 1000 mg/m^2^ with Dara-CyBorD administered I/V once every other week [90]. A subsequent study evaluating organ response in the same cohort (European modification stage I-IIIa AL amyloidosis) revealed that all nine patients with evaluable kidney involvement via 24 h urine protein achieved organ response, with five patients responding within 2 months of treatment initiation [91]. Additionally, eight out of nine renal evaluable patients treated with CAEL-101 and Dara-CyBorD demonstrated a ≥30% decrease in proteinuria based on 24 h protein measurement [92]. Currently, two phase III trials (NCT04504825 and NCT04512235) are underway, evaluating the efficacy of CAEL-101 in Mayo stage IIIa and IIIb cardiac AL-amyloidosis patients [93,94]. 

Birtamimab (i.e., NEOD001), an anti-light chain antibody 2A4 derivative, binds to the amyloid light chain and facilitates its clearance through antibody-dependent phagocytosis [95]. The first-in-human study evaluating the use of birtamimab in 27 AL-amyloidosis patients demonstrated its overall safety and tolerability, recommending monthly IV infusions at a dose of 24 mg/kg. Secondary endpoint analysis revealed that 57% and 60% of cardiac and renal patients met the organ response criteria [96]. However, the subsequent phase IIb PRONTO study failed to meet its primary endpoint (all-cause mortality and cardiac response rate) and was prematurely halted. The phase III VITAL (cardiac AL amyloidosis) study was also terminated following a futility analysis [97]. In the RAIN study, patients with renal AL amyloidosis who achieved a hematological response to prior therapy but continued to exhibit renal impairment were randomized to receive 24 mg/kg IV birtamimab or placebo. This trial was prematurely stopped by Prothena Biotech after their other two randomized trials failed to show a statistical organ response benefit to birtamimab use [98]. However, a recent post hoc analysis of 77 Mayo stage IV patients from the VITAL study cohort demonstrated a significant survival benefit in the group receiving birtamimab at 9 months post-treatment initiation compared to placebo (74% vs. 49%, respectively; HR, 0.413; 95% CI, 0.191–0.895; log-rank *p* = 0.021) [99]. These findings justified the confirmatory, phase 3, randomized, double-blinded, placebo-controlled AFFIRM-AL clinical trial of birtamimab in patients with Mayo stage IV AL amyloidosis patients (NCT04973137). Clinical trials evaluating birtamimab use in late-stage renal amyloidosis patients are also warranted.

Other anti-fibril agents include dezamizumab and the monoclonal antibody AT-02. AT-02 is a full-length, humanized, recombinant immunoglobulin 1 (IgG1)-like glycoprotein monoclonal antibody (mAb) engineered by fusion with the PAR p5R peptide. AT-02 is part of a group of new pan-amyloid agents designed for amyloid removal [100]. AT-02 is undergoing a multicenter, international, three-part Phase 1 study (NCT05521022). Dezamizumab is a monoclonal antibody that triggers the immunotherapeutic clearance of amyloid fibrils after serum amyloid P (SAP), a protein covering circulating amyloid fibrils, is depleted by miridesap (parenteral) or GSK3039294 (oral) [101,102]. Although early results with dezamizumab revealed favorable tolerability and safety results, clinical trials assessing this drug for cardiac amyloidosis have been terminated due to a lack of efficacy, attributed to the limited cardiac uptake observed through immune-positron emission tomography [103]. In a retrospective cohort study, doxycycline disrupted the formation of amyloid fibrils in mice and improved hematologic response and survival in patients with cardiac AL amyloidosis [104,105]. However, it failed to demonstrate a progression-free survival or cardiac response benefit in a multicenter, open-label, randomized, controlled trial involving patients with Mayo 2004 stage II-III AL amyloidosis [106].

### 5.4. Hematopoietic Stem Cell Transplant

ASCT remains a cornerstone in the management of transplant-eligible AL-amyloidosis patients [107]. The evolution of patient-tailored approaches for ASCT in AL amyloidosis involves carefully considering patient-related factors, including performance status and disease-specific factors, particularly the extent of cardiac involvement and heart failure. This individualized approach has been made feasible by identifying factors associated with poorer ASCT outcomes. These factors include cardiac involvement beyond stage II, higher heart failure status (NYHA > II), elevated NT-ProBNP levels (>5000 ng/L), multiple-organ involvement, frailty, fluid overload, arrhythmias, hypotension, genetics indicative of high risk, lack of hematologic response, elevated creatinine levels (>2), reduced melphalan dosage leading to early relapse, and undergoing transplants in lower-volume centers (less than four per year) [108,109]. However, one study reported that elevated Troponin I elevations and NT-ProBNP levels >5000 ng/L were not adverse prognostic indicators in patients with cardiac amyloidosis [110]. The risk of fluid overload, hypotension, and arrhythmias—factors associated with significant mortality risk in ASCT patients—is notably elevated in the presence of pre-existing nephrotic syndrome and underlying congestive heart failure. Batalini et al. demonstrated the efficacy of HDM-ASCT in inducing hematologic CR and prolonging OS in patients with AL amyloidosis or monoclonal immunoglobulin deposition disease with ESRD on dialysis [111]. This highlights the potential for renal transplantation in individuals achieving durable hematologic responses. Through factor screening using the consensus criteria for ASCT eligibility [112], 20–30% of patients now meet the eligibility criteria for ASCT, leading to significantly reduced treatment-related mortality (TRM) [108,109,113,114,115,116]. 

Renal function is a crucial consideration in therapeutic decision-making, especially in ASCT. The current consensus criteria for ASCT eligibility include physiological parameters, such as a creatinine clearance of more than 50%, while elevated serum creatinine (>2.0) has been associated with shorter survival [108,117]. Patients on hemodialysis or peritoneal dialysis are not excluded from consideration if they meet other eligibility criteria. Havasi et al., validated the use of the Pavia renal staging system in AL amyloidosis to predict ESRD progression in ASCT patients [36,118]. Although baseline renal involvement did not significantly impact OS, the median survival was 39 months after ESRD diagnosis. Patients diagnosed early with less advanced renal damage were less likely to progress to ESRD long-term. Conversely, a study of 408 ASCT patients from 1996 to 2010 found that 17.6% (72 patients) required dialysis. Notably, the timing of renal failure relative to transplant significantly impacted overall survival; patients needing dialysis within 30 days of ASCT experienced the highest treatment-related mortality (44.4%) [119]. In a multivariate analysis, serum albumin of <2.5 g/dL and eGFR <40 mL/min.1.73 m^2^ were associated with starting dialysis within 30 days of ASCT. These results suggest that screening for these parameters may help identify patients at high risk of transplant-related mortality. 

Consolidation therapy post-ASCT—with an emphasis on achieving deep responses—is considered for AL amyloidosis patients falling short of a VGPR. However, the potential benefits must be balanced with the toxicity risks, optimal response levels, and choice of agents. Two phase II trials explored the use of thalidomide/dexamethasone and bortezomib/dexamethasone consolidation post-ASCT [120,121]. A retrospective study demonstrated improved progression-free survival in patients with less than VGPR who received post-ASCT consolidation therapy [122]. A recent phase 3, randomized, placebo-controlled clinical trial in ASCT-eligible multiple myeloma patients demonstrated that the addition of daratumumab to an induction and a consolidation regimen of bortezomib, lenalidomide, and dexamethasone, as well as to lenalidomide maintenance therapy, significantly improved progression-free survival at 48 months (HR, 0.42; 95% CI, 0.30 to 0.59; *p* < 0.001), as well as secondary endpoints of complete response (87.9 vs. 70.1%, *p* < 0.001) and MRD negativity (75.2% vs. 47.5%, *p* < 0.001) [123]. Whether adding daratumumab to ASCT-eligible AL amyloidosis for consolidation has similar benefits remains to be elucidated. 

### 5.5. Dialysis and Kidney Transplant

The need for dialysis does not dictate a specific treatment regimen, but it does necessitate dose adjustments to the used medications. Bortezomib, for instance, typically does not require dose adjustment; however, this should be administered post-dialysis. In contrast, melphalan does require dose adjustments to 140 mg/m^2^ and may lead to unpredictable hematologic toxicity in cases of severe renal dysfunction. Among the IMiDs, only lenalidomide requires dose adjustment. While daratumumab can be safely administered to patients with severe renal dysfunction or those undergoing dialysis, data from relapsed/refractory AL amyloidosis suggest that I/V daratumumab’s efficacy may be reduced in patients with nephrotic-range proteinuria (discussed above). 

Pallidini et al., reported that the majority of cases of ESRD in patients with AL amyloidosis occur in the first 2 years, with 17–30% of survivors requiring dialysis by that time [36]. Beyond this period, the risk of dialysis is just a few percent per year. These findings are comparable to those of a study of 752 patients with renal AL amyloidosis, showing that 13% of patients progressed to ESRD within a median time of 26.8 months [124]. In a multicenter observational study spanning from 1987 to 2020 and covering 237 AL amyloidosis patients, The International Kidney and Monoclonal Gammopathy Research Group found that renal transplantation was significantly associated with prolonged overall patient survival and allograft survival, particularly for patients who achieved at least a hematological VGPR [125]. The median overall survival following renal transplantation was 8.6 years, and notably longer for patients achieving a VGPR or better than patients who achieved less than VGPR (9 vs. 6.8 years, respectively). Graft survival was also better in the CR + VGPR group than the less than VGPR group (8.3 vs. 5.7 years, respectively), coupled with a lower amyloid recurrence frequency (16% vs. 37%, respectively) and longer time to recurrence (median time not achieved vs. 10 years, respectively) [125]. Although no additional benefit could be demonstrated for CR patients compared to VGPR, this may be due to the ESRD affecting serum FLC levels, making it challenging to distinguish CR patients from VGPR. Furthermore, although hematologic relapse occurred in 29% of patients, most patients (87%) did not experience graft loss through treatment [125]. On this basis, it is recommended that kidney transplant be recommended for AL amyloidosis ESRD patients who have achieved at least a VGPR and who do not exhibit significant cardiac involvement and/or other contraindications to renal transplant [125,126]. 

## 6. Conclusions

Our continually expanding treatment armamentarium against AL amyloidosis—exemplified by the use of Dara-CyBorD for induction and venetoclax for t(11:14) AL amyloidosis—has led to rapid and deep hematological and organ responses, significantly prolonging patient survival. Novel treatment strategies on the horizon, such as anti-BCMA CAR-T cells/bispecific antibodies, anti-fibril antibodies, their combination therapy, and novel combinations with venetoclax for t(11:14) AL amyloidosis (although an overall survival benefit has not yet been shown), also present new avenues for intervention in both newly diagnosed and relapsed/refractory AL amyloidosis cases. A holistic and personalized management approach, rooted in a deeper understanding of the disease and its responses to diverse therapeutic interventions, will be crucial for optimizing patient outcomes and improving the overall management of amyloidosis across various patient populations. Testing the efficacy of novel immunotherapeutic strategies—including anti-fibril medications and anti-BCMA immunotherapies—to achieve deep renal responses and facilitate amyloid removal from tissues remains an area of investigation for future research. 

## Figures and Tables

**Table 1:** Diagnostic workup of AL amyloidosis.

Diagnostic Tests to Evaluate Systemic AL Amyloidosis
Assessment for monoclonal gammopathy Serum protein electrophoresis with immunofixation 24 h urine protein collection for electrophoresis with immunofixation Free light chain assay 2. Histological confirmation via Congo-red staining (Gold Standard) Abdominal fat pad aspiration Bone marrow biopsy Minor salivary gland biopsy Involved organ biopsy 3. Amyloid Typing Laser microdissection and mass spectroscopy-based proteomic analysis (preferred) Immunofluorescence vs. immunohistochemistry vs. electron microscopy 4. Other laboratory investigations (to be carried out while awaiting biopsy) cTNT, Troponin I, NT-proBNP, or BNP 24 h urine for albumin Complete blood count Renal profile and basic chemistry Alkaline phosphatase FISH on bone marrow biopsy 5. Imaging Studies EKG, transthoracic echocardiography, cardiac MRI ^99m^Tc-PYP/DPD/HMDP scan 6. Others Assessment of comorbidities If MGUS is absent, genetic DNA tests for hereditary forms

**Table 2:** Staging systems for AL amyloidosis based on cardiac involvement.

System	Risk Factors	Stages		Hazard Ratio for Death (95% CI)
Mayo 2004 [31]	(1) NT-proBNP ≥ 332 ng/L or (BNP ≥ 81 ng/L) (2) cTnT ≥ 0.035 mcg/L or (cTnI ≥ 0.1 mcg/L) or (hsTnT ≥ 50 ng/L)	Stage I	no risk factor	Reference
		Stage II	1 risk factor	2.5 (1.9–3.5)
		Stage III	2 risk factors	6.7 (5.0–9.1)
Mayo 2004 with European modifications [32]	Same risk factors as Mayo 2004	Stage I	no risk factor	Reference
		Stage II	1 risk factor	2.5 (1.9–3.5)
		Stage IIIa	2 risk factors and NT-proBNP < 8500 ng/L or BNP < 700 ng/L	4.9 (3.6–6.8)
		Stage IIIb	2 risk factors and NT-proBNP ≥ 8500 ng/L or BNP ≥ 700 ng/L	11.1 (8.1–15.4)
Mayo 2012 [33]	(1) NT-proBNP ≥ 1800 ng/L or (BNP ≥ 400 ng/L) (2) cTnT ≥ 0.025 mcg/L or (hsTnT ≥ 40 ng/L) (3) dFLC ≥ 180 mg/L	Stage I	no risk factors	Reference
		Stage II	1 risk factor	1.7 (1.2–2.3)
		Stage III	2 risk factors	4.1 (3.1–5.5)
		Stage IV	3 risk factors	6.3 (4.8–8.3)
Boston University [34]	(1) Cardiac TnI > 0.1 ng/mL (2) BNP > 81 pg/mL	Stage I	no risk factor	Median OS > 12 years
		Stage II	1 risk factor	Median OS 9.4 years
		Stage IIIa	2 risk factors and BNP < 700 pg/mL	Median OS 4.3 years
		Stage IIIb	2 risk factors and BNP > 700 pg/mL	Median OS 1 year

**Table 3:** Criteria for renal staging, response, and progression.

		Palladini et al. (2014) [36]	Kastritis et al. (2017) [37]	Basset et al. (2022) [38]
Staging	Stage I	eGFR > 50 mL/min and Proteinuria < 5 g/24 h	24h UPr/eGFR ratio < 30	eGFR > 50 mL/min and UACR < 3600 mg/g
	Stage II	eGFR < 50 mL/min or Proteinuria > 5 g/24 h	24h UPr/eGFR ratio 30–99	eGFR < 50 mL/min or UACR ≥ 3600 mg/g
	Stage III	eGFR < 50 mL/min and Proteinuria > 5 g/24 h	24h UPr/eGFR ratio ≥ 100	eGFR < 50 mL/min and UACR ≥ 3600 mg/g
Response		≥30% decrease in 24 h UPr or 24 h UPr < 0.5 g in the absence of kidney progression	≥25% decrease in 24 h UPr/eGFR ratioOR ratio < 100(If initially >100)	≥30% decrease in UACR in the absence of kidney progression
Progression		≥25% decrease in eGFR	≥25% increase in 24 h UPr/eGFR ratio OR ratio ≥ 100	≥25% decrease in eGFR

**Table 4:** Hematologic response assessment.

Response	Criteria
Complete	Both criteria must be met: (1) Negative serum and urine immunofixation (2) Either a FLC ratio within the reference range or the uninvolved FLC concentration is greater than involved FLC concentration with or without an abnormal FLC ratio
VGPR	Reduction in the dFLC to <40 mg/L
Partial	>50% reduction in the dFLC
No response	Less than a PR
Progression	From CR: any detectable M-protein or abnormal FLC ratio (light chain must double) From PR: 50% increase in serum M protein to >0.5 g/dL or 50% increase in urine M protein to >200 mg/day (a visible peak must be present) FLC increase of 50% to >100 mg/L

CR, complete response; FLC, free light chain; dFLC, difference between involved and uninvolved FLC; M-protein, monoclonal protein; PR, partial response; VGPR, very good partial response.

**Table 5:** Graded organ response criteria.

Organ	Category	Criteria
**Heart** [45]	Cardiac complete response (CarCR)	Nadir NT-proBNP ≤ 350 pg/mL or BNP ≤ 80 pg/mL
	Cardiac very good partial response (CarVGPR)	>60% reduction in NT-proBNP/BNP from baseline level not meeting CarCR
	Cardiac partial response (CarPR)	31–60% reduction in NT-proBNP from baseline level not meeting CarCR
	Cardiac no response (CarNR)	≤30% reduction in NT-proBNP from baseline level
**Renal** [46]	Renal complete response (RenCR)	Nadir proteinuria ≤ 200 mg/24-h
	Renal very good partial response (RenVGPR)	>60% reduction in proteinuria from baseline level not meeting RenCR
	Renal partial response (RenPR)	31–60% reduction in proteinuria from baseline level not meeting RenCR
	Renal no response (RenNR)	≤30% reduction in proteinuria from baseline level

## Data Availability

No new data were created or analyzed in this study. Data sharing is not applicable to this article.

## References

[1] Bianchi G., Kumar S. (2020). Systemic Amyloidosis Due to Clonal Plasma Cell Diseases. Hematol. Oncol. Clin. North. Am..

[2] Desport E., Bridoux F., Sirac C., Delbes S., Bender S., Fernandez B., Quellard N., Lacombe C., Goujon J.M., Lavergne D. (2012). Al amyloidosis. Orphanet J. Rare Dis..

[3] Merlini G., Dispenzieri A., Sanchorawala V., Schönland S.O., Palladini G., Hawkins P.N., Gertz M.A. (2018). Systemic immunoglobulin light chain amyloidosis. Nat. Rev. Dis. Primers.

[4] Dember L.M. (2006). Amyloidosis-associated kidney disease. J. Am. Soc. Nephrol..

[5] Gurung R., Li T. (2022). Renal Amyloidosis: Presentation, Diagnosis, and Management. Am. J. Med..

[6] Muchtar E., Dispenzieri A., Magen H., Grogan M., Mauermann M., McPhail E.D., Kurtin P.J., Leung N., Buadi F.K., Dingli D. (2021). Systemic amyloidosis from A (AA) to T (ATTR): A review. J. Intern. Med..

[7] Dima D., Mazzoni S., Anwer F., Khouri J., Samaras C., Valent J., Williams L. (2023). Diagnostic and Treatment Strategies for AL Amyloidosis in an Era of Therapeutic Innovation. JCO Oncol. Pract..

[8] Oubari S., Naser E., Papathanasiou M., Luedike P., Hagenacker T., Thimm A., Rischpler C., Kessler L., Kimmich C., Hegenbart U. (2021). Impact of time to diagnosis on Mayo stages, treatment outcome, and survival in patients with AL amyloidosis and cardiac involvement. Eur. J. Haematol..

[9] Lousada I., Comenzo R.L., Landau H., Guthrie S., Merlini G. (2015). Light Chain Amyloidosis: Patient Experience Survey from the Amyloidosis Research Consortium. Adv. Ther..

[10] Dispenzieri A., Buadi F., Kumar S.K., Reeder C.B., Sher T., Lacy M.Q., Kyle R.A., Mikhael J.R., Roy V., Leung N. (2015). Treatment of Immunoglobulin Light Chain Amyloidosis: Mayo Stratification of Myeloma and Risk-Adapted Therapy (mSMART) Consensus Statement. Mayo Clin. Proc..

[11] Schulman A., Connors L.H., Weinberg J., Mendelson L.M., Joshi T., Shelton A.C., Sanchorawala V. (2020). Patient outcomes in light chain (AL) amyloidosis: The clock is ticking from symptoms to diagnosis. Eur. J. Haematol..

[12] Muchtar E., Gertz M.A., Kyle R.A., Lacy M.Q., Dingli D., Leung N., Buadi F.K., Hayman S.R., Kapoor P., Hwa Y.L. (2019). A Modern Primer on Light Chain Amyloidosis in 592 Patients with Mass Spectrometry-Verified Typing. Mayo Clin. Proc..

[13] Novak L., Cook W.J., Herrera G.A., Sanders P.W. (2004). AL-amyloidosis is underdiagnosed in renal biopsies. Nephrol. Dial. Transplant..

[14] Satoskar A.A., Burdge K., Cowden D.J., Nadasdy G.M., Hebert L.A., Nadasdy T. (2007). Typing of amyloidosis in renal biopsies: Diagnostic pitfalls. Arch. Pathol. Lab. Med..

[15] Sethi S., Theis J.D., Leung N., Dispenzieri A., Nasr S.H., Fidler M.E., Cornell L.D., Gamez J.D., Vrana J.A., Dogan A. (2010). Mass spectrometry-based proteomic diagnosis of renal immunoglobulin heavy chain amyloidosis. Clin. J. Am. Soc. Nephrol..

[16] Leung N., Nasr S.H., Sethi S. (2012). How I treat amyloidosis: The importance of accurate diagnosis and amyloid typing. Blood.

[17] van G., Hazenberg B.P., Bijzet J., van Rijswijk M.H. (2006). Diagnostic accuracy of subcutaneous abdominal fat tissue aspiration for detecting systemic amyloidosis and its utility in clinical practice. Arthritis Rheumatol..

[18] Muchtar E., Dispenzieri A., Lacy M.Q., Buadi F.K., Kapoor P., Hayman S.R., Gonsalves W., Warsame R., Kourelis T.V., Chakraborty R. (2017). Overuse of organ biopsies in immunoglobulin light chain amyloidosis (AL): The consequence of failure of early recognition. Ann. Med..

[19] Soares S.M., Fervenza F.C., Lager D.J., Gertz M.A., Cosio F.G., Leung N. (2008). Bleeding complications after transcutaneous kidney biopsy in patients with systemic amyloidosis: Single-center experience in 101 patients. Am. J. Kidney Dis..

[20] Fermand J.P., Bridoux F., Kyle R.A., Kastritis E., Weiss B.M., Cook M.A., Drayson M.T., Dispenzieri A., Leung N. (2013). How I treat monoclonal gammopathy of renal significance (MGRS). Blood.

[21] Rosner M.H., Jhaveri K.D., McMahon B.A., Perazella M.A. (2021). Onconephrology: The intersections between the kidney and cancer. CA Cancer J. Clin..

[22] Jonathan W., Emily M., Alan S., Dustin P., Heidel R.E., Ronald L., Stephen K. (2022). Final Results of the First-In-Human Study of the Amyloid-Reactive Peptide 124I-p5+14, (Iodine[124I] Evuzamitide; AT-01) For the Detection of Systemic Amyloidosis. J. Nucl. Med..

[23] Cuddy S.A.M., Bravo P.E., Falk R.H., El-Sady S., Kijewski M.F., Park M.A., Ruberg F.L., Sanchorawala V., Landau H., Yee A.J. (2020). Improved Quantification of Cardiac Amyloid Burden in Systemic Light Chain Amyloidosis: Redefining Early Disease?. JACC Cardiovasc. Imaging.

[24] Nodoushani A., El-Sady M.S., Park M.-A., Castilloveitia G.L., Falk R.H., Di Carli M.F., Kijewski M.F., Dorbala S. (2021). Reproducibility and Repeatability of Assessment of Myocardial Light Chain Amyloidosis Burden Using 18F-Florbetapir PET/CT. J. Nucl. Cardiol..

[25] Eric C.E., El-Sady M.S., Marie F.K., Yiu Ming K., Sophia J., Frederick L.R., Vaishali S., Heather L., Andrew J.Y., Giada B. (2019). Early Detection of Multiorgan Light-Chain Amyloidosis by Whole-Body ^18^F-Florbetapir PET/CT. J. Nucl. Med..

[26] Gillmore J.D., Maurer M.S., Falk R.H., Merlini G., Damy T., Dispenzieri A., Wechalekar A.D., Berk J.L., Quarta C.C., Grogan M. (2016). Nonbiopsy Diagnosis of Cardiac Transthyretin Amyloidosis. Circulation.

[27] Garcia-Pavia P., Rapezzi C., Adler Y., Arad M., Basso C., Brucato A., Burazor I., Caforio A.L.P., Damy T., Eriksson U. (2021). Diagnosis and treatment of cardiac amyloidosis. A position statement of the European Society of Cardiology Working Group on Myocardial and Pericardial Diseases. Eur. J. Heart Fail..

[28] Castano A., Haq M., Narotsky D.L., Goldsmith J., Weinberg R.L., Morgenstern R., Pozniakoff T., Ruberg F.L., Miller E.J., Berk J.L. (2016). Multicenter Study of Planar Technetium 99m Pyrophosphate Cardiac Imaging: Predicting Survival for Patients With ATTR Cardiac Amyloidosis. JAMA Cardiol..

[29] Hanna M., Ruberg Frederick L., Maurer Mathew S., Dispenzieri A., Dorbala S., Falk Rodney H., Hoffman J., Jaber W., Soman P., Witteles Ronald M. (2020). Cardiac Scintigraphy With Technetium-99m-Labeled Bone-Seeking Tracers for Suspected Amyloidosis. J. Am. Coll. Cardiol..

[30] Rauf M.U., Hawkins P.N., Cappelli F., Perfetto F., Zampieri M., Argiro A., Petrie A., Law S., Porcari A., Razvi Y. (2023). Tc-99m labelled bone scintigraphy in suspected cardiac amyloidosis. Eur. Heart J..

[31] Dispenzieri A., Gertz M.A., Kyle R.A., Lacy M.Q., Burritt M.F., Therneau T.M., Greipp P.R., Witzig T.E., Lust J.A., Rajkumar S.V. (2004). Serum cardiac troponins and N-terminal pro-brain natriuretic peptide: A staging system for primary systemic amyloidosis. J. Clin. Oncol..

[32] Wechalekar A.D., Schonland S.O., Kastritis E., Gillmore J.D., Dimopoulos M.A., Lane T., Foli A., Foard D., Milani P., Rannigan L. (2013). A European collaborative study of treatment outcomes in 346 patients with cardiac stage III AL amyloidosis. Blood.

[33] Kumar S., Dispenzieri A., Lacy M.Q., Hayman S.R., Buadi F.K., Colby C., Laumann K., Zeldenrust S.R., Leung N., Dingli D. (2012). Revised prognostic staging system for light chain amyloidosis incorporating cardiac biomarkers and serum free light chain measurements. J. Clin. Oncol. Off. J. Am. Soc. Clin. Oncol..

[34] Lilleness B., Ruberg F.L., Mussinelli R., Doros G., Sanchorawala V. (2019). Development and validation of a survival staging system incorporating BNP in patients with light chain amyloidosis. Blood.

[35] Dittrich T., Kimmich C., Hegenbart U., Schönland S.O. (2020). Prognosis and Staging of AL Amyloidosis. Acta Haematol..

[36] Palladini G., Hegenbart U., Milani P., Kimmich C., Foli A., Ho A.D., Rosin M.V., Albertini R., Moratti R., Merlini G. (2014). A staging system for renal outcome and early markers of renal response to chemotherapy in AL amyloidosis. Blood.

[37] Kastritis E., Gavriatopoulou M., Roussou M., Migkou M., Fotiou D., Ziogas D.C., Kanellias N., Eleutherakis-Papaiakovou E., Panagiotidis I., Giannouli S. (2017). Renal outcomes in patients with AL amyloidosis: Prognostic factors, renal response and the impact of therapy. Am. J. Hematol..

[38] Basset M., Milani P., Ferretti V.V., Nuvolone M., Foli A., Benigna F., Nanci M., Bozzola M., Ripepi J., Sesta M. (2022). Prospective urinary albumin/creatinine ratio for diagnosis, staging, and organ response assessment in renal AL amyloidosis: Results from a large cohort of patients. Clin. Chem. Lab. Med..

[39] Visram A., Al Saleh A.S., Parmar H., McDonald J.S., Lieske J.C., Vaxman I., Muchtar E., Hobbs M., Fonder A., Hwa Y.L. (2020). Correlation between urine ACR and 24-h proteinuria in a real-world cohort of systemic AL amyloidosis patients. Blood Cancer J..

[40] Palladini G., Dispenzieri A., Gertz M.A., Kumar S., Wechalekar A., Hawkins P.N., Schönland S., Hegenbart U., Comenzo R., Kastritis E. (2012). New Criteria for Response to Treatment in Immunoglobulin Light Chain Amyloidosis Based on Free Light Chain Measurement and Cardiac Biomarkers: Impact on Survival Outcomes. J. Clin. Oncol..

[41] Kastritis E., Kostopoulos I.V., Theodorakakou F., Fotiou D., Gavriatopoulou M., Migkou M., Tselegkidi M.I., Roussou M., Papathoma A., Eleutherakis-Papaioakovou E. (2020). Next generation flow cytometry for MRD detection in patients with AL amyloidosis. Amyloid.

[42] Milani P., Basset M., Russo F., Foli A., Merlini G., Palladini G. (2017). Patients with light-chain amyloidosis and low free light-chain burden have distinct clinical features and outcome. Blood.

[43] Dittrich T., Bochtler T., Kimmich C., Becker N., Jauch A., Goldschmidt H., Ho A.D., Hegenbart U., Schönland S.O. (2017). AL amyloidosis patients with low amyloidogenic free light chain levels at first diagnosis have an excellent prognosis. Blood.

[44] Comenzo R.L., Reece D., Palladini G., Seldin D., Sanchorawala V., Landau H., Falk R., Wells K., Solomon A., Wechalekar A. (2012). Consensus guidelines for the conduct and reporting of clinical trials in systemic light-chain amyloidosis. Leukemia.

[45] Muchtar E., Dispenzieri A., Wisniowski B., Palladini G., Milani P., Merlini G., Schönland S., Veelken K., Hegenbart U., Geyer S.M. (2023). Graded Cardiac Response Criteria for Patients With Systemic Light Chain Amyloidosis. J. Clin. Oncol. Off. J. Am. Soc. Clin. Oncol..

[46] Muchtar E., Wisniowski B., Palladini G., Milani P., Merlini G., Schönland S., Veelken K., Hegenbart U., Dispenzieri A., Kumar S. (2021). Graded Renal Response Criteria for Light Chain (AL) Amyloidosis. Blood.

[47] Kastritis E., Palladini G., Minnema M.C., Wechalekar A.D., Jaccard A., Lee H.C., Sanchorawala V., Gibbs S., Mollee P., Venner C.P. (2021). Daratumumab-Based Treatment for Immunoglobulin Light-Chain Amyloidosis. N. Engl. J. Med..

[48] Chakraborty R., Bhutani D., Mapara M., Reshef R., Maurer M.S., Radhakrishnan J., Lentzsch S. (2023). Reduced Early Mortality with Daratumumab-Based Frontline Therapy in Systemic AL Amyloidosis- Experience from the Columbia Amyloidosis Multidisciplinary Program. Blood.

[49] Staron A., Zheng L., Doros G., Connors L.H., Mendelson L.M., Joshi T., Sanchorawala V. (2021). Marked progress in AL amyloidosis survival: A 40-year longitudinal natural history study. Blood Cancer J..

[50] Muchtar E., Gertz M.A., Kumar S.K., Lacy M.Q., Dingli D., Buadi F.K., Grogan M., Hayman S.R., Kapoor P., Leung N. (2017). Improved outcomes for newly diagnosed AL amyloidosis between 2000 and 2014: Cracking the glass ceiling of early death. Blood.

[51] Kimmich C.R., Terzer T., Benner A., Dittrich T., Veelken K., Carpinteiro A., Hansen T., Goldschmidt H., Seckinger A., Hose D. (2020). Daratumumab for systemic AL amyloidosis: Prognostic factors and adverse outcome with nephrotic-range albuminuria. Blood.

[52] Lecumberri R., Krsnik I., Askari E., Sirvent M., González-Pérez M.S., Escalante F., Pradillo V., Tamariz L.E., Cánovas V., Alegre A. (2020). Treatment with daratumumab in patients with relapsed/refractory AL amyloidosis: A multicentric retrospective study and review of the literature. Amyloid.

[53] Hughes M.S., Lentzsch S. (2023). Safety and Efficacy of Subcutaneous Daratumumab in Systemic AL Amyloidosis. Ther. Clin. Risk Manag..

[54] Kim K., Phelps M.A. (2023). Clinical Pharmacokinetics and Pharmacodynamics of Daratumumab. Clin. Pharmacokinet..

[55] Palladini G., Milani P., Foli A., Basset M., Russo F., Perlini S., Merlini G. (2018). Presentation and outcome with second-line treatment in AL amyloidosis previously sensitive to nontransplant therapies. Blood.

[56] Wechalekar A.D., Cibeira M.T., Gibbs S.D., Jaccard A., Kumar S., Merlini G., Palladini G., Sanchorawala V., Schönland S., Venner C. (2022). Guidelines for non-transplant chemotherapy for treatment of systemic AL amyloidosis: EHA-ISA working group. Amyloid.

[57] Palladini G., Milani P. (2023). Diagnosis and Treatment of AL Amyloidosis. Drugs.

[58] Sanchorawala V. (2019). Delay treatment of AL amyloidosis at relapse until symptomatic: Devil is in the details. Blood Adv..

[59] Wechalekar A.D., Sanchorawala V. (2022). Daratumumab in AL amyloidosis. Blood.

[60] Sanchorawala V., Sarosiek S., Schulman A., Mistark M., Migre M.E., Cruz R., Sloan J.M., Brauneis D., Shelton A.C. (2020). Safety, tolerability, and response rates of daratumumab in relapsed AL amyloidosis: Results of a phase 2 study. Blood.

[61] Roussel M., Merlini G., Chevret S., Arnulf B., Stoppa A.M., Perrot A., Palladini G., Karlin L., Royer B., Huart A. (2020). A prospective phase 2 trial of daratumumab in patients with previously treated systemic light-chain amyloidosis. Blood.

[62] Staron A., Burks E.J., Szalat R.E., Sanchorawala V. (2023). Prospective Evaluation of Measurable Residual Disease (MRD) and Sustainability of the Response in Patients with Systemic AL Amyloidosis Treated with Daratumumab. Blood.

[63] Kimmich C.R., Terzer T., Benner A., Hansen T., Carpinteiro A., Dittrich T., Veelken K., Jauch A., Huhn S., Basset M. (2021). Daratumumab, lenalidomide, and dexamethasone in systemic light-chain amyloidosis: High efficacy, relevant toxicity and main adverse effect of gain 1q21. Am. J. Hematol..

[64] Rosenbaum C., Liedtke M., Christos P., Kim H., Pogonowski K., Agudo N., Barroso B., Shelton A., Reilly S., Gadbois C. (2023). Daratumumab, Pomalidomide and Dexamethasone (DPd) in Relapsed/ Refractory Light Chain Amyloidosis Previously Exposed to Daratumumab. Blood.

[65] Dumas B., Yameen H., Sarosiek S., Sloan J.M., Sanchorawala V. (2020). Presence of t(11;14) in AL amyloidosis as a marker of response when treated with a bortezomib-based regimen. Amyloid.

[66] Bochtler T., Hegenbart U., Kunz C., Granzow M., Benner A., Seckinger A., Kimmich C., Goldschmidt H., Ho A.D., Hose D. (2015). Translocation t(11;14) is associated with adverse outcome in patients with newly diagnosed AL amyloidosis when treated with bortezomib-based regimens. J. Clin. Oncol..

[67] Bianchi G., Zhang Y., Comenzo R.L. (2021). AL Amyloidosis: Current Chemotherapy and Immune Therapy Treatment Strategies: JACC: CardioOncology State-of-the-Art Review. JACC CardioOncol..

[68] Premkumar V.J., Lentzsch S., Pan S., Bhutani D., Richter J., Jagannath S., Liedtke M., Jaccard A., Wechalekar A.D., Comenzo R. (2021). Venetoclax induces deep hematologic remissions in t(11;14) relapsed/refractory AL amyloidosis. Blood Cancer J..

[69] Lebel E., Kastritis E., Palladini G., Milani P., Theodorakakou F., Aumann S., Lavi N., Shargian L., Magen H., Cohen Y. (2023). Venetoclax in Relapse/Refractory AL Amyloidosis-A Multicenter International Retrospective Real-World Study. Cancers.

[70] Theodorakakou F., Spiliopoulou V., Fotiou D., Roussou M., Malandrakis P., Ntanasis-Stathopoulos I., Migkou M., Eleutherakis Papaiakovou E., Kanellias N., Papanikolaou A. (2022). Outcomes of Patients with AL Amyloidosis after Failure of Daratumumab-Based Therapy. Blood.

[71] Orland M., Dima D., Ullah F., Awada H., Basali D., Faiman B.M., Scott C., Mazzoni S., Williams L.S., Samaras C.J. (2023). Outcomes of Venetoclax-Based Therapy in Patients with Daratumumab-Refractory t(11;14) Positive Light Chain Amyloidosis. Blood.

[72] Shah N., Chari A., Scott E., Mezzi K., Usmani S.Z. (2020). B-cell maturation antigen (BCMA) in multiple myeloma: Rationale for targeting and current therapeutic approaches. Leukemia.

[73] Yu B., Jiang T., Liu D. (2020). BCMA-targeted immunotherapy for multiple myeloma. J. Hematol. Oncol..

[74] Khwaja J., Bomsztyk J., Bygrave C., Forbes A., Durairaj S., Fernandes S., Taylor J., Paterson P., Brearton G., Crawley C. (2023). High Responses Rates with Single Agent Belantamab Mafodotin in Relapsed Systemic AL Amyloidosis. Blood.

[75] Lebel E., Vainstein V., Milani P., Palladini G., Shragai T., Lavi N., Magen H., Miri A., Avivi Mazza I., Gatt M.E. (2023). Belantamab Mafodotin in Relapse/Refractory AL Amyloidosis- Real-Life Multi-Center Experience. Blood.

[76] Zhang Y., Godara A., Pan S., Toskic D., Mann H., Sborov D., Comenzo R., Kansagra A. (2022). Belantamab mafodotin in patients with relapsed/refractory AL amyloidosis with myeloma. Ann. Hematol..

[77] van de Donk N.W., Usmani S.Z., Yong K. (2021). CAR T-cell therapy for multiple myeloma: State of the art and prospects. Lancet Haematol..

[78] Scheller L., Tebuka E., Rambau P.F., Einsele H., Hudecek M., Prommersberger S.R., Danhof S. (2024). BCMA CAR-T cells in multiple myeloma–ready for take-off?. Leuk. Lymphoma.

[79] Oliver-Caldes A., Jiménez R., Español-Rego M., Cibeira M.T., Ortiz-Maldonado V., Quintana L.F., Castillo P., Guijarro F., Tovar N., Montoro M. (2021). First report of CART treatment in AL amyloidosis and relapsed/refractory multiple myeloma. J. Immunother. Cancer.

[80] Kfir-Erenfeld S., Asherie N., Grisariu S., Avni B., Zimran E., Assayag M., Sharon T.D., Pick M., Lebel E., Shaulov A. (2022). Feasibility of a Novel Academic BCMA-CART (HBI0101) for the Treatment of Relapsed and Refractory AL Amyloidosis. Clin. Cancer Res..

[81] Moreau P., Garfall A.L., van de Donk N.W.C.J., Nahi H., San-Miguel J.F., Oriol A., Nooka A.K., Martin T., Rosinol L., Chari A. (2022). Teclistamab in Relapsed or Refractory Multiple Myeloma. N. Engl. J. Med..

[82] Mann H., Toskic D., Pak K., Willard P., Fogaren T., Comenzo R. (2023). Teclistamab Induces Rapid Responses in Patients with Relapsed/Refractory AL Amyloidosis. Blood.

[83] Forgeard N., Elessa D., Carpinteiro A., Belhadj K., Minnema M.C., Roussel M., Huart A., Javaugue V., Pascal L., Royer B. (2024). Teclistamab in relapsed or refractory AL amyloidosis, a multinational retrospective case series. Blood.

[84] Esteghamat S., Ahlawat D., Raiker S., Batonick M., Quarta C.C., Usmani-Brown S. (2022). C11-1F4 Binds and Promotes Clearance of Light Chain Fibrils By Phagocytosis. Blood.

[85] Costello M., Park N., Neill A., Wong E., Müller K., Proffitt A., Huynh R., Rao V., Sun F., Bozzi A. (2023). Cael-101 Enhances the Clearance of Light Chain Fibrils and Intermediate Aggregates By Phagocytosis. Blood.

[86] Edwards C.V., Rao N., Bhutani D., Mapara M., Radhakrishnan J., Shames S., Maurer M.S., Leng S., Solomon A., Lentzsch S. (2021). Phase 1a/b study of monoclonal antibody CAEL-101 (11-1F4) in patients with AL amyloidosis. Blood.

[87] Khouri J., Anwer F., Samaras C.J., Mejia Garcia A.V., Koc O.N., Faiman B.M., Hamilton K., Mathur S., Scott C., Stefunek K. (2020). Safety, Tolerability and Efficacy of Cael-101 in AL Amyloidosis Patients Treated on a Phase 2, Open-Label, Dose Selection Study to Evaluate the Safety and Tolerability of Cael-101 in Patients with AL Amyloidosis. Blood.

[88] Edwards C.V., Gould J., Langer A.L., Mapara M., Radhakrishnan J., Maurer M.S., Raza S., Mears J.G., Wall J., Solomon A. (2017). Interim analysis of the phase 1a/b study of chimeric fibril-reactive monoclonal antibody 11-1F4 in patients with AL amyloidosis. Amyloid.

[89] Valent J., Liedtke M., Zonder J.A., Manwani R., Udata C., Ianus J., Tripptree J., Catini J., Quarta C.C. (2023). Safety and Tolerability of Cael-101, an Anti-Amyloid Monoclonal Antibody, Combined with Anti-Plasma Cell Dyscrasia Therapy in Patients with Light-Chain Amyloidosis: 24-Month Results of a Phase 2 Study. Blood.

[90] Valent J., Silowsky J., Kurman M.R., Daniel E., Jobes J., Harnett M., Ziola M., Roviwong J., Fada S., Spector M. (2020). Cael-101 Is Well-Tolerated in AL Amyloidosis Patients Receiving Concomitant Cyclophosphamide-Bortezomib-Dexamethasone (CyborD): A Phase 2 Dose-Finding Study (NCT04304144). Blood.

[91] Valent J., Silowsky J., Kurman M.R., Daniel E., Jobes J., Harnett M., Fada S., Spector M., Stefunek K., Staley H. (2021). Updated Organ Response Results of an Open-Label Study to Evaluate the Safety and Tolerability of Cael-101 in Patients with AL Amyloidosis Receiving Anti-Plasma Cell Therapy. Blood.

[92] Valent J., Liedtke M., Zonder J.A., Tulchinskiy M., Udata C., Ramirez G., Szymaniak K., Catini J., Quarta C.C. (2022). 1-Year Results from a Phase 2 Study to Determine Safety and Tolerability of Treating Patients with Light-Chain (AL) Amyloidosis with Cael-101, an Anti-Amyloid Monoclonal Antibody, Combined with Anti-Plasma Cell Dyscrasia. Blood.

[93] Wechalekar A.D., Silowsky J., Daniel E., Harnett M., Spector M., Sobolov S.B., Quarta C.C., Kurman M.R., Tulchinskiy M., Bhattacharyya A. (2021). Cardiac Amyloid Reaching for Extended Survival (CARES): Study Design of Two Placebo-Controlled, Double-Blind, Randomized, International Phase 3 Trials Assessing Cael-101 in Patients with Mayo Stage Iiia or Stage IIIb AL Amyloidosis. Blood.

[94] Liedtke M., Palladini G., Molina M.A., Kastritis E., Ianus J., Catini J., Quarta C.C., Wechalekar A.D. (2023). Enrolling patients in Cardiac Amyloid Reaching for Extended Survival (CARES) trials: Two placebo-controlled, double-blind, randomized, international phase 3 trials assessing CAEL-101 in patients with Mayo stage IIIa or stage IIIb AL amyloidosis. J. Clin. Oncol..

[95] Renz M., Torres R., Dolan P.J., Tam S.J., Tapia J.R., Li L., Salmans J.R., Barbour R.M., Shughrue P.J., Nijjar T. (2016). 2A4 binds soluble and insoluble light chain aggregates from AL amyloidosis patients and promotes clearance of amyloid deposits by phagocytosis. Amyloid.

[96] Gertz M.A., Landau H., Comenzo R.L., Seldin D., Weiss B., Zonder J., Merlini G., Schönland S., Walling J., Kinney G.G. (2016). First-in-Human Phase I/II Study of NEOD001 in Patients with Light Chain Amyloidosis and Persistent Organ Dysfunction. J. Clin. Oncol..

[97] Gertz M.A., Cohen A.D., Comenzo R.L., Du Mond C., Kastritis E., Landau H.J., Libby E.N., Liedtke M., Merlini G., Sanchorawala V. (2019). Results of the Phase 3 VITAL Study of NEOD001 (Birtamimab) Plus Standard of Care in Patients with Light Chain (AL) Amyloidosis Suggest Survival Benefit for Mayo Stage IV Patients. Blood.

[98] Varga C., Lentzsch S., Comenzo R.L. (2018). Beyond NEOD001 for systemic light-chain amyloidosis. Blood.

[99] Gertz M.A., Cohen A.D., Comenzo R.L., Kastritis E., Landau H.J., Libby E.N., Liedtke M., Sanchorawala V., Schönland S., Wechalekar A. (2023). Birtamimab plus standard of care in light-chain amyloidosis: The phase 3 randomized placebo-controlled VITAL trial. Blood.

[100] Emdin M., Morfino P., Crosta L., Aimo A., Vergaro G., Castiglione V. (2023). Monoclonal antibodies and amyloid removal as a therapeutic strategy for cardiac amyloidosis. Eur. Heart J. Suppl..

[101] Richards D.B., Cookson L.M., Barton S.V., Liefaard L., Lane T., Hutt D.F., Ritter J.M., Fontana M., Moon J.C., Gillmore J.D. (2018). Repeat doses of antibody to serum amyloid P component clear amyloid deposits in patients with systemic amyloidosis. Sci. Transl. Med..

[102] Richards D., Bamford M., Liefaard L., Haque N., Lewis G., Storey J., Fernando D., Kumar S., Thompson D., Holmes D.S. (2020). Identification, preclinical profile, and clinical proof of concept of an orally bioavailable pro-drug of miridesap. Br. J. Pharmacol..

[103] Wechalekar A., Antoni G., Al Azzam W., Bergström M., Biswas S., Chen C., Cheriyan J., Cleveland M., Cookson L., Galette P. (2022). Pharmacodynamic evaluation and safety assessment of treatment with antibodies to serum amyloid P component in patients with cardiac amyloidosis: An open-label Phase 2 study and an adjunctive immuno-PET imaging study. BMC Cardiovasc. Disord..

[104] Ward J.E., Ren R., Toraldo G., SooHoo P., Guan J., O’Hara C., Jasuja R., Trinkaus-Randall V., Liao R., Connors L.H. (2011). Doxycycline reduces fibril formation in a transgenic mouse model of AL amyloidosis. Blood J. Am. Soc. Hematol..

[105] Wechalekar A.D., Whelan C. (2017). Encouraging impact of doxycycline on early mortality in cardiac light chain (AL) amyloidosis. Blood Cancer J..

[106] Shen K.-N., Fu W.-J., Wu Y., Dong Y.-J., Huang Z.-X., Wei Y.-Q., Li C.-R., Sun C.-Y., Chen Y., Miao H.-L. (2022). Doxycycline Combined With Bortezomib-Cyclophosphamide-Dexamethasone Chemotherapy for Newly Diagnosed Cardiac Light-Chain Amyloidosis: A Multicenter Randomized Controlled Trial. Circulation.

[107] Bal S., Estrada-Merly N., Costa L.J., Qazilbash M.H., Kumar S., D’Souza A. (2023). Outcomes of t(11;14) light chain (AL) amyloidosis after autologous stem cell transplantation: Benchmark for new therapies. Blood Cancer J..

[108] Sidiqi M.H., Aljama M.A., Buadi F.K., Warsame R.M., Lacy M.Q., Dispenzieri A., Dingli D., Gonsalves W.I., Kumar S., Kapoor P. (2018). Stem Cell Transplantation for Light Chain Amyloidosis: Decreased Early Mortality Over Time. J. Clin. Oncol..

[109] Gustine J.N., Staron A., Szalat R.E., Mendelson L.M., Joshi T., Ruberg F.L., Siddiqi O., Gopal D.M., Edwards C.V., Havasi A. (2022). Predictors of hematologic response and survival with stem cell transplantation in AL amyloidosis: A 25-year longitudinal study. Am. J. Hematol..

[110] Gertz M.A., Lacy M.Q., Dispenzieri A., Kumar S.K., Dingli D., Leung N., Hogan W.J., Buadi F.K., Hayman S.R. (2013). Refinement in patient selection to reduce treatment-related mortality from autologous stem cell transplantation in amyloidosis. Bone Marrow Transplant..

[111] Batalini F., Econimo L., Quillen K., Sloan J.M., Sarosiek S., Brauneis D., Havasi A., Stern L., Dember L.M., Sanchorawala V. (2018). High-Dose Melphalan and Stem Cell Transplantation in Patients on Dialysis Due to Immunoglobulin Light-Chain Amyloidosis and Monoclonal Immunoglobulin Deposition Disease. Biol. Blood Marrow Transplant..

[112] Palladini G., Milani P., Merlini G. (2020). Management of AL amyloidosis in 2020. Hematol. Am Soc Hematol. Educ. Program.

[113] D’Souza A., Dispenzieri A., Wirk B., Zhang M.J., Huang J., Gertz M.A., Kyle R.A., Kumar S., Comenzo R.L., Peter Gale R. (2015). Improved Outcomes After Autologous Hematopoietic Cell Transplantation for Light Chain Amyloidosis: A Center for International Blood and Marrow Transplant Research Study. J. Clin. Oncol..

[114] Sidana S., Sidiqi M.H., Dispenzieri A., Buadi F.K., Lacy M.Q., Muchtar E., Dingli D., Hayman S.R., Gonsalves W.I., Kapoor P. (2019). Fifteen year overall survival rates after autologous stem cell transplantation for AL amyloidosis. Am. J. Hematol..

[115] Sanchorawala V., Sun F., Quillen K., Sloan J.M., Berk J.L., Seldin D.C. (2015). Long-term outcome of patients with AL amyloidosis treated with high-dose melphalan and stem cell transplantation: 20-year experience. Blood.

[116] Skinner M., Sanchorawala V., Seldin D.C., Dember L.M., Falk R.H., Berk J.L., Anderson J.J., O’Hara C., Finn K.T., Libbey C.A. (2004). High-dose melphalan and autologous stem-cell transplantation in patients with AL amyloidosis: An 8-year study. Ann. Intern. Med..

[117] Fuchida S.I., Kawamura K., Sunami K., Tsukada N., Fujii S., Ohkawara H., Usuki K., Wake A., Endo S., Ishiyama K. (2022). Retrospective Analysis of Autologous Stem Cell Transplantation for AL Amyloidosis: A Study from the Multiple Myeloma Working Group of the Japan Society for Hematopoietic Cell Transplantation. Transplant. Cell Ther..

[118] Havasi A., Stern L., Lo S., Sun F., Sanchorawala V. (2016). Validation of new renal staging system in AL amyloidosis treated with high dose melphalan and stem cell transplantation. Am. J. Hematol..

[119] Leung N., Kumar S.K., Glavey S.V., Dispenzieri A., Lacy M.Q., Buadi F.K., Hayman S.R., Dingli D., Kapoor P., Zeldenrust S.R. (2016). The impact of dialysis on the survival of patients with immunoglobulin light chain (AL) amyloidosis undergoing autologous stem cell transplantation. Nephrol. Dial. Transplant..

[120] Landau H., Hassoun H., Rosenzweig M.A., Maurer M., Liu J., Flombaum C., Bello C., Hoover E., Riedel E., Giralt S. (2013). Bortezomib and dexamethasone consolidation following risk-adapted melphalan and stem cell transplantation for patients with newly diagnosed light-chain amyloidosis. Leukemia.

[121] Cohen A.D., Zhou P., Chou J., Teruya-Feldstein J., Reich L., Hassoun H., Levine B., Filippa D.A., Riedel E., Kewalramani T. (2007). Risk-adapted autologous stem cell transplantation with adjuvant dexamethasone +/− thalidomide for systemic light-chain amyloidosis: Results of a phase II trial. Br. J. Haematol..

[122] Al Saleh A.S., Sidiqi M.H., Sidana S., Muchtar E., Dispenzieri A., Dingli D., Lacy M.Q., Warsame R.M., Gonsalves W.I., Kourelis T.V. (2019). Impact of consolidation therapy post autologous stem cell transplant in patients with light chain amyloidosis. Am. J. Hematol..

[123] Sonneveld P., Dimopoulos M.A., Boccadoro M., Quach H., Ho P.J., Beksac M., Hulin C., Antonioli E., Leleu X., Mangiacavalli S. (2023). Daratumumab, Bortezomib, Lenalidomide, and Dexamethasone for Multiple Myeloma. N. Engl. J. Med..

[124] Pinney J.H., Lachmann H.J., Bansi L., Wechalekar A.D., Gilbertson J.A., Rowczenio D., Sattianayagam P.T., Gibbs S.D., Orlandi E., Wassef N.L. (2011). Outcome in renal Al amyloidosis after chemotherapy. J. Clin. Oncol..

[125] Havasi A., Heybeli C., Leung N., Angel-Korman A., Sanchorawala V., Cohen O., Wechalekar A., Bridoux F., Jaffer I., Gutgarts V. (2022). Outcomes of renal transplantation in patients with AL amyloidosis: An international collaboration through The International Kidney and Monoclonal Gammopathy Research Group. Blood Cancer J..

[126] Angel-Korman A., Stern L., Sarosiek S., Sloan J.M., Doros G., Sanchorawala V., Havasi A. (2019). Long-term outcome of kidney transplantation in AL amyloidosis. Kidney Int..

